# Dendritic versus somatic resonance

**DOI:** 10.1186/1471-2202-12-S1-P289

**Published:** 2011-07-18

**Authors:** Ekaterina A Zhuchkova, Susanne Schreiber

**Affiliations:** 1 Institute for Theoretical Biology, Humboldt University, Berlin, 10115, Germany; 2 Bernstein Center for Computational Neuroscience, Berlin, 10115, Germany

## 

Membrane-potential resonance characterizes the ability of a neuron to selectively respond to stimuli in a preferred frequency band. It has been associated with the occurrence of subthreshold membrane-potential oscillations (MPOs) and has been shown to be of functional relevance, as exemplified by the correlation of resonance frequencies with the spacing of grid fields in the entorhinal cortex [1] and the dependence of this spacing on the resonating H current [2].

Resonance arises from the interaction of passive and active membrane properties, usually requiring the presence of slowly-activating conductances that act as high-pass filters and are able to effectively oppose slow changes of the membrane potential. The distribution of slow conductances responsible for resonance (like H or M), however, can differ between the compartments within a neuron. In CA1 neurons, for example, it is known that the density of H channels increases by more than 60-fold from soma to dendrites and is largest in the distal parts of the dendritic tree [3]. Accordingly, resonance can also depend on the spatial localization within a cell. Still, cells are usually classified as either resonant or nonresonant on the basis of somatic injection of ZAP currents (sine-wave functions with a linear increase in frequency).

Here, we investigate to what extent and under which circumstances cells with dendritic resonance may be misclassified as nonresonant by somatic measurement of resonance properties. We use simple conductance-based multicompartmental models to analyze the effect of dendritic resonance on somatic input (and hence resonance estimates based on somatic recordings). We find that indeed, even a strong dendritic resonance may not be detectable with somatic ZAP protocols. The extent to which dendritic resonance is masked depends on neuronal morphology as well as the distribution of active conductances within the cell. In addition, we show that although dendritic resonance may not show up somatically, indirect consequences of dendritic resonance can affect the soma. In particular, MPOs of dendritic resonance-induced origin may propagate to the soma, leading to a situation where such cells when measured somatically do exhibit subthreshold MPOs in the apparent "absence" of resonance.

A local dendritic resonance filters dendritic inputs - even if it should not show up somatically - and is hence crucial for the flow of information in neuronal networks. It is therefore important to identify the circumstances under which dendritic resonance could be missed in somatic assessment of resonance properties.
